# Experimental Studies of the Effect of Microencapsulated PCM Slurry on the Efficiency of a Liquid Solar Collector

**DOI:** 10.3390/ma15134493

**Published:** 2022-06-25

**Authors:** Tadeusz Bohdal, Krzysztof Dutkowski, Marcin Kruzel

**Affiliations:** Department of Power Engineering, Faculty of Mechanical Engineering, Koszalin University of Technology, 75-453 Koszalin, Poland; krzysztof.dutkowski@tu.koszalin.pl

**Keywords:** phase change materials, reduced temperature, mPCM slurry, flat plate solar collector

## Abstract

A phase change material (PCM) is used as a substance filling in a heat store, due to the possibility of accumulating a significant amount of latent heat—the heat of phase transformation. Knowledge about the practical use of the working fluid, with the addition of a phase change substance, in heat exchange systems is limited The paper presents the results of preliminary research aimed at determining the possibility of using microencapsulated phase change material slurry (mPCM) as a working fluid in installations with a flat liquid solar collector, and the potential benefits as a result. The following were used as the working fluid during the tests: water (reference liquid), and a slurry of microencapsulated PCM. The mass fraction of mPCM in the working liquids is 4.3% and 8.6%, respectively. The research was carried out in laboratory conditions, in the range of radiation intensity G = 270–880 W/m^2^. The mass flux of each of the three working fluids in the collector is 30 kg/h, 40, kg/h, 60 kg/h, and 80 kg/h. Two main advantages of using mPCM as an additive to the working liquid are found: 1. in the entire range of thermal radiation intensity, the increase in the thermal efficiency of the collector fed with slurries is 4% with 4.3% mPCM in the slurry, and 6% with 8.6% mPCM in the slurry (for $\dot{m}$ = 80 kg/h); 2. the slurry is characterized by a lower temperature at the outlet from the collector as compared to the water with the same thermal and flow parameters, which reduces heat losses to the environment both from the collector and other elements of the installation, as a result of excessive heating of the working liquid.

## 1. Introduction

Solar collectors are constantly modified to better serve people by meeting their domestic hot water needs. Various modifications to their design led to an increase in their efficiency, in order to obtain more solar energy in the form of useful heat. Some of them have widespread use. At the moment, further modifications do not bring such spectacular results. Currently, new technologies are used to intensify heat transfer while obtaining solar energy. An interesting solution is the use of thermal energy storage techniques. Solar energy can be stored as a change in the internal energy of the material as sensible heat, latent heat, chemical heat, or a combination of the above. Adsorption and release of heat take place during the phase change from solid to liquid, or liquid to gas, or vice versa. For this purpose, phase change materials (PCM) were successfully used. The miniaturization of the encapsulation process enables the application of microencapsulated phase change materials (mPCM). Microencapsulation is the process by which very small particles or droplets are surrounded or covered by a foil [1]. The interest in using mPCM slurry media as thermal storage and heat transfer fluids is increasing and, thus, leading to an enhancement in energy usage. For this purpose, numerous studies are carried out on the use of phase change materials [2,3,4,5,6].

## 2. The Current State of Knowledge

Photothermal conversion and energy storage using phase change materials are now applied in many processes and technologies. Some studies describe the use of PCM in solar systems, for example, as tank filling in a part of the coupled PCM–solar thermal collector system [7,8,9], the filling of evacuated tube solar collectors [10,11], or the fabrication and combination of composite phase change material wallboard [12] with the solar thermal system. PCM is also used in a solar distillation system [13], or in the production of sustainable water from novel-designed tubular solar still [14]. It is found that achieving energy storage benefits the phase change material, and reduces the energy fluctuations, and means the solar thermal energy is stored during the daytime and released in the absence of sunlight. The disadvantage of heat storage with PCM is that its low thermal conductivity makes the heat transfer process difficult when the PCM is in a solid state. One of the methods of improving the thermal conductivity of PCM is adding substances with a high thermal conductivity to it, e.g., in the form of nanoparticles of substances with a high thermal conductivity coefficient [15,16]. Another way to improve heat transfer in PCM-based warehouses is to encapsulate PCM. This makes it possible to initiate a phase transformation of PCM in each of the closed volumes, and in addition, the capsule material increases heat conduction in the store. Increasing the number of capsules by reducing their volume leads to a situation where they reach a size of the order of micro or even nanometers. In this case, the working fluid no longer washes the encapsulated PCM material, but carries it with it. This gave rise to another group (after ice emulsions and slurries) of working fluids–micro/nano-encapsulated PCM slurries (mPCM/nPCM slurry). Currently, the mPCM slurry is used as a working fluid for cooling photovoltaic panels [17,18,19,20,21,22]. It is shown that it is possible to receive significant amounts of heat from PV panels while maintaining a low, practically unchanged temperature of the working liquid. The mPCM slurry can be used as a working liquid in the evacuated heat pipe solar collector [23], filling the heat store [24], and as a substance for direct absorption of heat radiation [25]. There are not many published research results on the use of mPCMS in flat liquid collectors. Most papers here describe the results of computer modeling of heat transfer [26,27,28,29,30,31,32]. The presented simulation results clearly show that the use of mPCM slurry as a working fluid in flat solar collectors may increase their efficiency from several to several dozen percent. These reports require verification by means of a physical experiment. Also, promising results of my own, and preliminary experimental studies published in [33] carried out by the authors of the study, confirm the need for further research in this field.

The review of the current state of knowledge shows that there is a clear shortage in the results of experimental research on the use of mPCM as liquid heat transfer media in flat plate solar collectors systems. The paper presents the results of our own experimental research on the influence of mass fraction of mPCM in the working fluid on the thermal efficiency of a flat solar collector. Water, 10% wt., and 20% wt. aqueous solution of the product, under the trade name MICRONAL^®^ 5428 X (Moraine, OH, USA), were used. As the product contains 43% mPCM, the mass fraction of mPCM in the working liquid is 4.3% and 8.6%, respectively. The obtained characteristics are presented, and the coefficients of the Hottel–Whillier equation are given.

## 3. Experiment

### 3.1. The Test Facility

**Figure 1:**
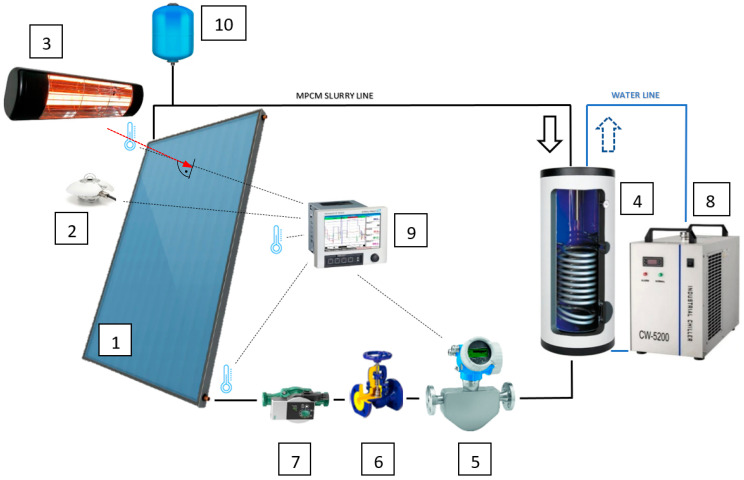
Schematic diagram of the experimental stand: 1. flat plate solar collector; 2. pyranometer; 3. infrared heater; 4. spiral coil heat exchanger; 5. Coriolis-type mass flow meter; 6. regulating valve; 7. circulation pump; 8. chiller; 9. recording device; 10. mPCM leveling vessel.

A schematic diagram of the experimental stand is presented in Figure 1. The main component of the experimental stand was a flat plate liquid solar collector (1) KSH-2.0 by Kospel (Koszalin, Poland), with the aperture area A = 1.98 m^2^.

**Figure 2:**
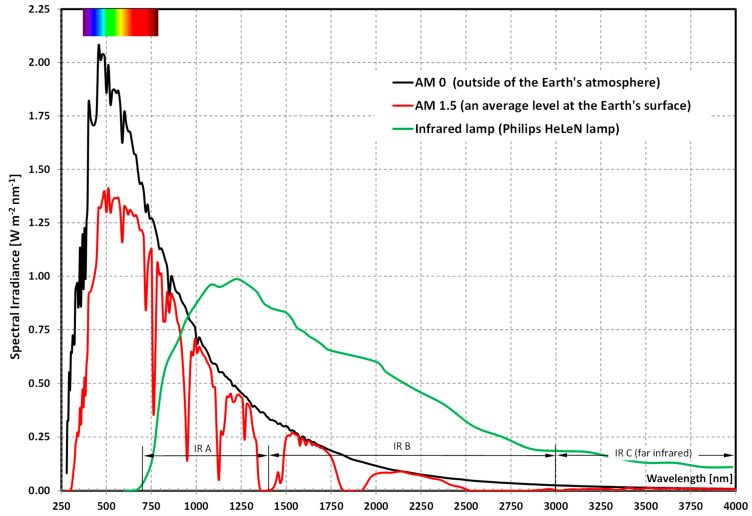
The Philips HeLeN filament spectrum of radiation vs. the spectrum of solar radiation.

Three radiation intensity-regulated infrared heaters (3), parallel to the collector, were installed at a 2 m distance from the front surface of the collector. Phillips HeLeN (Diez/Lahn, Germany) filaments are used in the radiators. The spectrum of radiation is presented vs. the spectrum of solar radiation (Figure 2). Taking into account that the spectrum of the filament radiation differs to the spectrum of solar radiation, it was not possible to compare the results of authors own experimental research with the results published by other authors. Therefore, the results of own research during the collector’s operation with mPCM slurry were compared with the results obtained by the authors during the tests carried out under analogous conditions, using distilled and demineralized water for the reference liquids.

The total radiation intensity reaching the collector surface was measured with a secondary standard CMP11 pyranometer by Kipp and Zonen (2) (Delft, The Netherlands). The radiation intensity was quantified on the basis of the arithmetic mean of 18 measurement points, evenly distributed over the entire surface of the collector. Under laboratory conditions, the maximum value of total irradiance of I = 1000 W/m^2^ was recorded. The average radiation intensity was determined with an accuracy of 4.33 W/m^2^.

The centrifugal pump YONOS PICO (Wilo, Dortmund, Germany) (7) generated the movement of the working medium through the collector in the measuring section, and then through a spiral coil of the heat exchanger (4) inside 100 l tank SW-100 Termo Max by KOSPEL (Koszalin, Poland). The slurry transferred the thermal energy obtained in the collector to the intermediate liquid cooled with a chilled water unit THAEY NF 109/t ASP0 by RHOSS (Codroipo, Italy) (8). The cooling water flowed through the exchanger as the coil’s surroundings. The cooled slurry was passed through a Coriolis mass flow meter (5), (Promass 80A by Endress-Hauser (Wien, Austria)). The device ensures measurement accuracy of ±0.15% of the measured value. The mPCM slurry with the set flow rate flowed into the pump suction port through the control valve (6).

Ambient temperature and mPCM slurry at the collector inlet and collector outlet were measured with K-type thermocouples individually calibrated (in the range of 10 °C–80 °C) using a standard glass thermometer with an elementary scale of 0.02 °C. The measurement error of the thermocouple oscillates in the range of ±0.2 K. Temperature, flow, and radiation intensity were archived by the RSG45 Memograph recorder (9) by Endress and Hauser (Wien, Austria). 

### 3.2. Working Fluid

**Figure 3:**
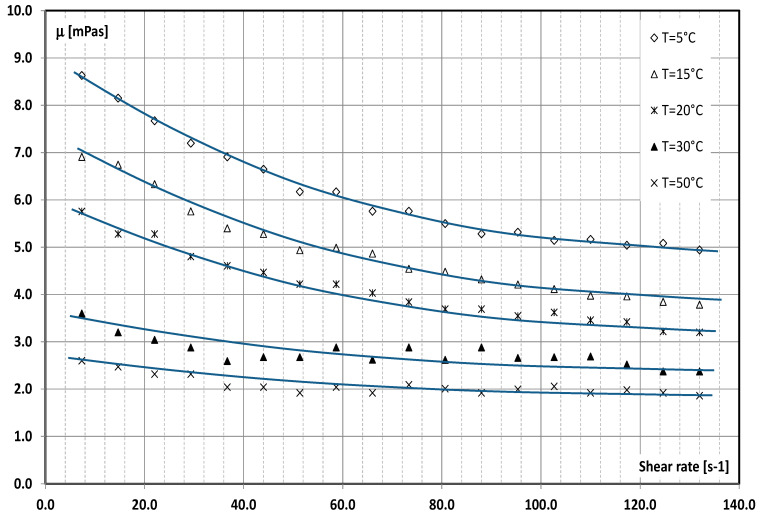
The viscosities of 8.6% mPCM slurries vs. shear rates at different temperatures.

In the tests, distilled and demineralized water (standard liquid) and two aqueous mPCM slurries (MICRONAL^®^ 5428) were used as the working liquids. The main component of the product was paraffin wax microencapsulated in a highly cross-linked polymethylmethacrylate polymer wall, and water. The mean peak of the melting point of paraffin is 28 °C, and the heat of fusion is 160 kJ/kg. The mean particle size of mPCM is 1–5 μm. The mass fraction of mPCM is, respectively, 4.3 and 8.6%. The thermodynamic and physicochemical properties of mPCM slurry used during the research were published by the authors. The mass fraction of mPCM is, respectively, 4.3 and 8.6%. The authors published the thermodynamic and physicochemical properties of mPCM slurry used during the research. The density [34], heat of fusion and specific heat [35], the heat conduction coefficient [36], and the viscosity of the slurry [37] were determined. Figure 3 shows the characteristics of the viscosity of the slurry with a concentration of 8.6% mPCM (used in the tests) as a function of temperature and shear rate.

### 3.3. Data Reduction

The experiment was carried out in a steady state. For each irradiance: G = 270 W/m^2^; 520 W/m^2^; 650 W/m^2^; and 880 W/m^2^ (for water) and G = 280 W/m^2^; 490 W/m^2^; 720 W/m^2^; and 880 W/m^2^ (for mPCM slurries), a series of measurements were performed at different flow rates of the working liquid (30 kg/h; 40 kg/h; 60 kg/h; and 80 kg/h). To determine the influence of the mass fraction of mPCM (4.3% wt. and 8.6% wt.) on the collector’s parameters, the temperature of the working medium at the collector inlet (T_in_ = 10 °C–27 °C) was maintained, and different temperatures of the working medium at the collector outlet, for the same experimental conditions, were influenced by the physical properties of the working medium. A constant ambient temperature of 23 ± 0.5 °C was ensured.

The heat absorbed by the heat transfer fluid (HTF) is determined according to formula:

(1) $$\dot{Q}=\dot{m}\cdot c_{p}\cdot \left(T_{i}-T_{o}\right),$$

where *T_i_* and *T_o_* are the water temperature at the inlet and outlet of the collector [K], respectively, *m* is the mass flux of the water [kg/s], and *c_p_* is the specific heat of the water [J/(kgK)].

**Figure 4:**
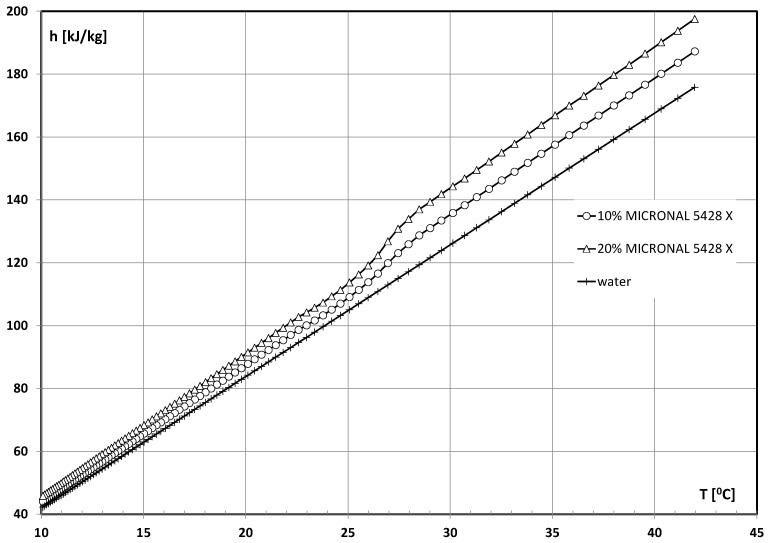
Values of working fluids’ specific enthalpy during the experiment.

The heat absorbed by mPCM slurry describes the formula:

(2) $$\dot{Q}=\dot{m}\cdot \left(h_{i}-h_{o}\right),$$

where *h_i_* and *h_o_* denote the specific enthalpy of the medium at the inlet and outlet of the collector, respectively [J/kg]. The specific heat of water is *c_p_* = 4.19 kJ/(kgK). The value of the specific enthalpy of mPCM slurry was determined experimentally. Data were obtained according to the so-called “Thermal delay method” [38]. The time-dependent cooling process was the basis for determining the specific heat. The reference fluid and mPCM slurry samples were initially heated to 50 °C. The instantaneous temperature that they reached at the same time during their cooling down to a temperature of 10 °C was then measured and compared. The specific heat of the slurry and the heat of phase changes were determined by converting the results. The procedure is described in detail in [35]. The influence of temperature on the specific enthalpy of mPCM slurry is presented in Figure 4.

Useful heat income of the working media in the collector can be expressed as the heat balance of the absorber plate:

(3) $${\dot{Q}}_{u}=A_{c}F_{R}\left[G\left(\tau \alpha \right)-U_{L}\left(T_{i}-T_{a}\right)\right],$$

where *G* is the irradiance value at the collector per unit area [W/m^2^], (τα) is the effective transmission and absorption coefficient, *F_R_* is the heat dissipation coefficient of the collector, and *U_L_* is the heat loss coefficient. Also, *T_a_*, *T_i_*, and *T_o_* are the ambient, media inlet, and outlet temperatures [K], respectively. The efficiency of flat plate solar collector was calculated according to the formula:

(4) $$\eta =\frac{{\dot{Q}}_{u}}{GA_{c}}=\frac{\dot{m}c_{p}\left(T_{o}-T_{i}\right)}{GA_{c}}.$$

Converting Equations (3) and (4), the formula Hottel–Whillier (5) is given for steady state calculations:

(5) $$\eta =F_{R}\left(\tau \alpha \right)-F_{R}U_{L}\frac{\left(T_{i}-T_{a}\right)}{G}.$$

**Figure 5:**
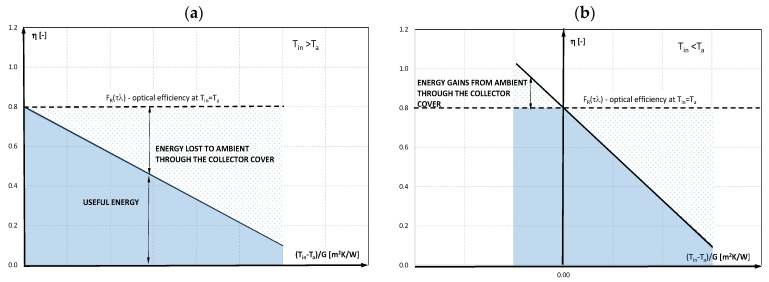
The diagram of thermal efficiency of solar collector: (**a**) T_in_ > T_a_ (only energy losses to ambient are possible); (**b**) T_in_ < T_a_ (energy gains and losses are possible).

When the performance data were collected under near normal irradiation conditions, where the (τα) value was constant, the *F_R_* and *U_L_* were constant within the testing conditions. The thermal efficiency can be plotted with a straight line against the related values of the heat loss parameter, according to Equation (5), with the intersection of the optical efficiency or the energy absorbed parameter ($F_{R}\left(\tau \alpha \right)$), and the slope of the parameter describing energy removed ($-F_{R}U_{L}$) [38]. The thermal efficiency diagram is presented in Figure 5.

The characteristics of the solar collector presented in the literature relate to cases when the temperature of the working liquid at the entrance to the solar collector is higher than the ambient temperature, and after the working liquid passes through the collector it further increases, so it is consistent with the diagram presented in Figure 5a. The mPCM concentrate used in the research is characterized by the fact that PCM undergoes a phase change at a temperature of about 25 °C, and the ambient temperature in the laboratory room was *T_a_*~23 °C. In order to clearly capture the influence of the PCM phase transformation on the temperature of the working medium leaving the collector and the collector’s efficiency, it was decided to supply it with a cooling liquid with a temperature of *T_i_* << *T_a_*. Hence, the characteristics obtained during the experimental tests lie in the area shown in Figure 5b.

## 4. Results of Experimental Research

**Figure 6:**
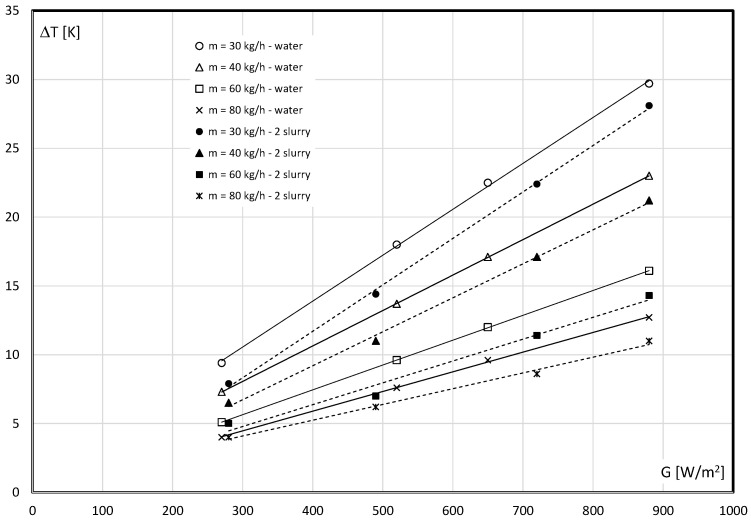
Influence of the irradiance and flow rate on the increase in temperature of the working liquid in the collector: (––––) water, (- - - -) 2nd slurry: 8.6% mPCM.

Figure 6 shows the influence of the mass flow rate of the working medium on its temperature increase. It is noted that the temperature difference of the medium between the inlet and outlet from the collector is smaller the greater the flow rate. Figure 6 shows the characteristics obtained during the flow of water in the form of continuous lines. The temperature increase of the 2nd slurry (8.6% mPCM: dashed line), at each mass flow rate and radiation intensity gave values a few degrees lower. These are the cases when the PCM flowing in the capsules undergoes a complete phase change, i.e., it is a solid at the entrance to the collector, and a liquid at the exit from the collector. On the basis of the tests performed, it is observed that the temperature of the slurry at the outlet from the collector, in similar cases, may be lower than the water temperature by about 5 °C. Lowering the temperature of the working liquid is essential during the operation of the solar system in winter conditions. Reducing the temperature difference between the air and the working fluid results in less heat loss to the environment.

**Figure 7:**
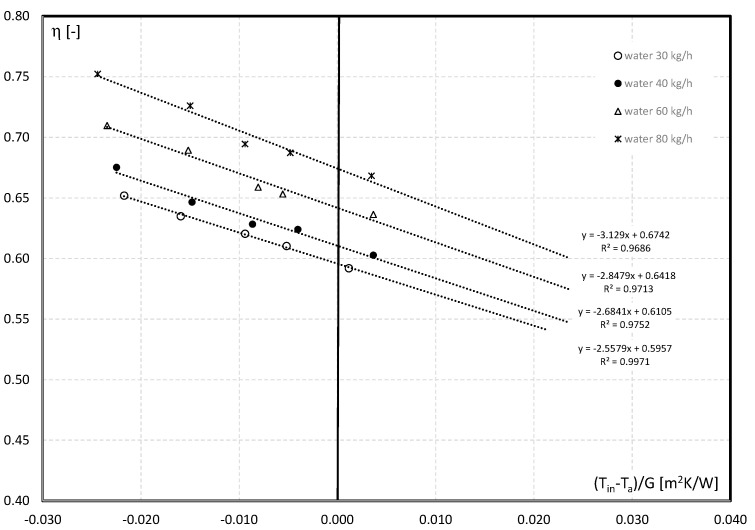
Effect of reduced temperature and water flow rate on thermal efficiency performance.

The experimental data calculated according to Equation (5) enables comparing the thermal efficiency of the collector as a function of the reduced temperature difference (*T_i_* − *T_a_*)/*G*. Figure 6 shows the influence of the water flow rate on the thermal efficiency of the collector. It is noted that the optical effectiveness *F_R_*(*τα*) of the water collector varies from 0.5957 (*m* = 30 kg/h) to 0.6742 (*m* = 80 kg/h). It is, therefore, concluded that the greater the water flow rate, the greater the collector’s efficiency, which is graphically reflected in characteristics (Figure 7). 

For all the considered values of the fluid flow, a decrease in the thermal efficiency of the collector is observed with the increase in the inlet temperature of the medium. This is due to the fact that the increase in temperature of the fluid entering the collector, and, thus, the absorber plates, causes more energy to be lost to the environment through convection, conduction, and radiation. Increasing the fluid flow rate results in a larger heat loss to the environment, and in a steeper slope of the efficiency curve and increase of *F_R_U_L_* value. A reflection of this fact in the experimental investigation is the change in the value of the *F_R_U_L_* coefficient from 2.5579 to 3.129. A similar effect is achieved, for example, by the use of Al_2_O_3_-Fe nanoparticles as an additive to water by Okonkwo et al. [39].

**Figure 8:**
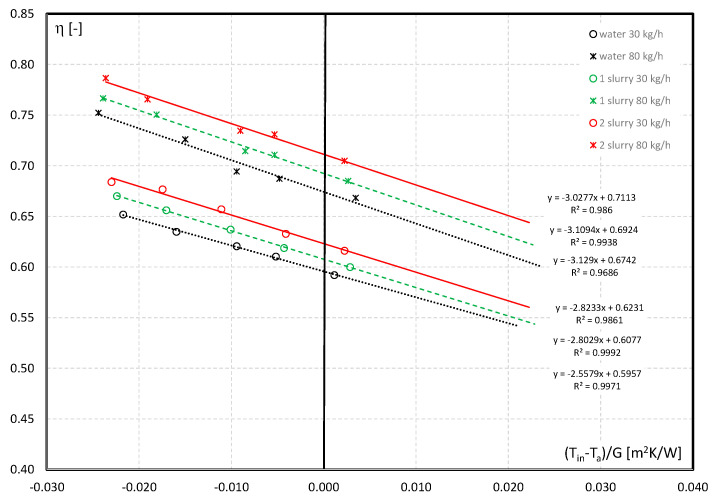
Influence of flow rate and mPCM addition on thermal efficiency performance of the collector vs. reduced temperature parameter: (·······) water; (- - - -) 1st slurry: 4.3% mPCM; (––––) 2nd slurry: 8.6% mPCM.

The effect of the addition of microcapsules containing PCM to water on the efficiency of the solar collector is shown in Figure 8. The characteristics relate to the flow of: water, 4.3% mPCM slurry (1st slurry), and 8.6% mPCM slurry (2nd slurry). A comparison of the characteristics obtained for two exemplary values of the working liquid flow (m = 30 kg/h and m = 80 kg/h) was made. It is noted that the use of slurries increases the thermal efficiency of the collector. It increases from 0.5957 to 0.6077 (4.3% mPCM) and to 0.6231 (8.6% mPCM) at a flow of 30 kg/h, and from 0.6742 to 0.6924 (4.3% mPCM) and to 0.7113 (8.6% mPCM). This means an increase of 2% and 4%for the lower flow, and 3% and 6% for the higher working fluid flow, respectively. MPCM slurries are characterized by a much higher heat transfer rate than of carrier fluid at a certain velocity. 

The increase in flow rate results in more intense flow turbulence. The chaotic movement of liquid particles also causes chaotic movement of solid particles–PCM microcapsules, which break the boundary layer in the heat exchange zone. The effect of breaking the boundary layer by the solid particles in the slurry is more intense than the effect caused by only the base liquid particles. Similar observations are made during experimental studies of heat transfer in straight sections of pipes described in the works.

## 5. Summary and Conclusions

The heat efficiency tests of the flat plate solar collector were carried out with the use of a new type of working liquid. Using a slurry of microencapsulated PCM as a working fluid was carried out. The mPCM content in the base liquid is 0% (“pure” reference liquid), 4.3%, and 8.6%. The research was carried out in the range of radiation intensity G = 270–880 W/m^2^. The mass flux of working fluids is 30 kg/h, 40, kg/h, 60 kg/h, and 80 kg/h. The results show that adding PCM microcapsules into the working fluid increases the thermal efficiency of the collector in the entire range of thermal radiation. The maximum increase in the thermal efficiency of the collector is 4% (4.3% mPCM; m = 80 kg/h) and 6% (8.6% mPCM; m = 80 kg/h). At the same time, the slurry is characterized by a lower temperature at the outlet from the collector. Therefore, it is possible to reduce the heat loss from the entire solar installation. The positive effect of adding microencapsulated PCM particles on the efficiency of energy conversion into a flat plate solar collector is demonstrated. Further studies are planned to determine the impact of the mass fraction of mPCM on the pumping power and the influence of the slurry with a higher mass fraction of mPCM on the efficiency of the collector. It is also planned to conduct research with the use of capsules containing PCM undergoing phase change at the operating temperature of typical solar installations.

## Data Availability

Not applicable.
